# Age-related sensitivity to task-related modulation of language-processing networks

**DOI:** 10.1016/j.neuropsychologia.2014.08.017

**Published:** 2014-10

**Authors:** Simon W. Davis, Jie Zhuang, Paul Wright, Lorraine K. Tyler

**Affiliations:** Centre for Speech, Language and the Brain, Department of Psychology, University of Cambridge, Cambridge CB2 3EB, UK

**Keywords:** Ageing, Language, Syntax, Functional Networks, ICA

## Abstract

It is widely assumed that cognitive functions decline with age and that these decrements are associated with age-related changes in patterns of functional activity. However, these functional changes may be due to age-related increased responsiveness to task demands and not to other cognitive processes on which neural and behavioural responses rely, since many ageing studies use task paradigms that may not be orthogonal to the cognitive function being investigated. Here we test this hypothesis in adults aged 20–86 years by combining measures of language comprehension, functional connectivity and neural integrity to identify functional networks activated in two language experiments with varying task demands. In one, participants listened to spoken sentences without performing an overt task (the natural listening condition) while in the other they performed a task in response to the same sentences. Using task-based ICA of fMRI, we identified a left-lateralised frontotemporal network associated with syntactic analysis, which remained consistently activated regardless of task demands. In contrast, in the task condition only a separate set of components showed task-specific activity in Opercular, Frontoparietal, and bilateral PFC. Only the PFC showed age-related increases in activation which, furthermore, was strongly mediated by grey matter health. These results suggest that, contrary to prevailing views, age-related changes in cognitive activation may be due in part to differential responses to task-related processes.

## Highlights

• Ageing effects on brain activity may be over-estimated due to demands of tasks.
• Age differences in PFC network activity were present only during an active task.
• This age-related increase is largely accounted for by grey matter morphology.
• Results suggest that age-related changes may be due to task-related processes.

## 1 Introduction

Ageing brains exhibit many changes in task-induced brain activity across the lifespan, most of which cannot be explained by physiological factors alone (D’Esposito et al., 1999, Kannurpatti et al., 2010). A central challenge in the study of healthy ageing is to understand how these neural changes relate to cognitive processes. One of the most common patterns associated with increasing age – increased bilateral prefrontal cortex (PFC) activity – has been observed across a wide range of tasks involving many cognitive functions (Dennis and Cabeza, 2008, Eyler et al., 2011). The functional relevance of this effect remains controversial (Grady, 2012), with some claiming that it may reflect either non-selective recruitment associated with decreased specialisation (Logan, Sanders, Snyder, Morris, & Buckner, 2002), or proactive cognitive control strategies formed in response to structural changes (Nyberg et al., 2010, Persson et al., 2011, Velanova et al., 2007). Support for both viewpoints has relied on the relationship between changing patterns of PFC activity and behavioural performance (Davis et al., 2008, Persson et al., 2006).

These findings raise a fundamental question: how do task requirements impact cognitive functions as we age? Previous studies have shown that task-related components generate additional functional activity in young participants over and above that involved in domain-specific processes (Cabeza et al., 2004, Meyer et al., 2000, Wright et al., 2011). Here we ask whether the relationship between task-related and domain-specific processes during language comprehension changes with age. We define here a *task effect* as the set of operations intrinsic to maintaining performance in the context of the experimental situation (Orne, 1962). Maintaining attention, storing arbitrary task heuristics, and manipulating information over short periods of time are demands ubiquitous in most studies of the ageing brain, yet form only a part of everyday life. Many everyday activities are highly practised and automatic, minimising the contribution of the kinds of task variables typically tested in studies of age-related changes in cognition. A growing number of studies have endeavoured to address this problem by using more realistic stimuli in fMRI studies. These novel paradigms include movie watching (Hasson, Furman, Clark, Dudai, & Davachi, 2008), story listening (Lerner, Honey, Silbert, & Hasson, 2011), and free recall of previously encoded personal events (St Jacques, Conway, Lowder, & Cabeza, 2011).

The focus on task effects poses particular problems for understanding the nature of the age-related changes in higher cognitive functions, since it is well-established that older adults show impairments in many of the mental operations intrinsic to performing experimental tasks. For example, older adults show performance deficits in disengaging with irrelevant stimuli (Hasher et al., 2007, Healey et al., 2008, Madden et al., 2007), switching between cognitive tasks (Jimura & Braver, 2010), and re-engaging after task interruption (Clapp, Rubens, Sabharwal, & Gazzaley, 2011), all of which have been associated with changes in bilateral PFC function. Previous ageing studies have addressed this problem by using a task-general approach to compare activation *across* different tasks in either empirical (Cabeza et al., 2004, Davis et al., 2008) or meta-analytic approaches (Spreng, Wojtowicz, & Grady, 2010); however, these strategies do not eliminate the problem since similar operations may be associated with many tasks.

In contrast to age-related decrements in such task-related cognitions, some cognitive functions are relatively preserved across the lifespan, especially when tested in more naturalistic contexts: implicit memory (Jennings & Jacoby, 1993), general knowledge (Hedden, Lautenschlager, & Park, 2005) and language comprehension (Radvansky & Copeland, 2001) each tap elements of everyday language function, and all show minimal effects of age when task demands are low and ecological validity is high. For example, Hedden et al. (2005) found that decrements in free recall performance are attenuated by general knowledge, suggesting that age-related increases in knowledge may compensate for age-related declines in performing a memory task. Similarly, Radvansky and Copeland (2001) showed that in contrast to more task-oriented memory for propositional information (which shows reliable age-related declines), both younger and older adults are similarly able to update event representations during natural story comprehension. These functions therefore represent ideal models in which to evaluate age-related effects of an experimental task on cognitive functions that, in their natural contexts, are highly automatised, but which in more experimental conditions show stronger age-related decrements.

In this study we investigated the effects of tasks on cognitive functions in ageing by focussing on a core component of human language—syntax. Much experimental research over the past 30 years has shown that syntactic analysis is highly automatised (Marslen-Wilson, 1975, Marslen-Wilson, 1987, Marslen-Wilson and Tyler, 1980) involving the rapid and obligatory mapping from speech sounds to lexical representations and the construction of syntactically structured sentential representations. These automatic processes are revealed in neural responses to spoken sentences even when participants are merely listening to the sentences without performing an explicit task (Crinion et al., 2003, Tyler and Marslen-Wilson, 2008). Furthermore, syntactic comprehension remains preserved across the lifespan and involves a reliable left-lateralised frontotemporal network (Dapretto and Bookheimer, 1999, Tyler et al., 2011), comprising a consistent set of regions including the left inferior gyrus (including Brodmann area 45) and the middle temporal gyrus (MTG), which can be characterised in young adults either with (Meyer et al., 2000) or without (Tyler et al., 2011) the involvement of an explicit task. Although considerable evidence suggests that this left frontotemporal network is engaged in the young in response to syntactic manipulations (Rodd et al., 2010, Turken and Dronkers, 2011, Tyler et al., 2013, Tyler et al., 2010), there is some evidence that bilateral frontal regions are activated in older adults. However, these increases in right PFC activity are not typically correlated with better syntactic performance (Antonenko et al., 2013, Tyler et al., 2010), raising the issue of what its functional role is. Thus, while a number of neuroimaging studies in older adults have reported bilateral PFC patterns of activation during language function, the relationship between the left frontotemporal language network and other task-related networks across the adult lifespan is unclear. One possibility – explored in the current paper – is that the additional right PFC activity in older people is related to the increased contribution of other task-related networks; our study therefore seeks to explicitly address under which conditions the bilateral frontal PFC network and the left frontotemporal language network are relevant to normal language function.

Independent component analysis (ICA) has proven to be very effective in isolating independent but overlapping functional networks present in a given fMRI dataset and provides information that augments the classical univariate approach (Beckmann and Smith, 2004, Calhoun et al., 2001). These advantages are particularly useful for cognitive neuroscience investigations of ageing, as differences in response time (Salthouse, 1996) and hemodynamic responsivity (Huettel et al., 2001, Velanova et al., 2003) may complicate and confound the interpretation of significant differences between age groups based on standard univariate approaches. In the current study we carried out an ICA-based functional connectivity analysis of fMRI data, combining information from two language studies: one in which participants performed an explicit task in response to sentential stimuli [*task condition*] and one in which they simply listened to the same sentences [*natural listening condition*]. This analysis allowed us to separate the neurocognitive networks that were stable between the two scanning studies, from networks that come online in response to performing an experimental task. This, in turn, allowed us to characterise the age-related changes in functional network activity occurring during the task in relation to those occurring during natural listening.

## 2 Material and methods

### 2.1 Participants

The participants consisted of 50 healthy, native British English speakers across a broad range of ages (20–86 years). Participants were tested in two scanning experiments – *task* and *natural listening* – and were evenly divided between the two experiments (task: 12 females; natural listening: 13 females), with equivalent distributions of subjects across the lifespan in each experiment (Mann–Whitney *U*=284.0, *p*=0.58). There was no overlap in the participant pools between the *task* and *natural listening* experiments. They were all right-handed with no history of neurological illness or head injury and free of psychiatric illness or psychoactive medication for at least 6 months prior to scanning. No participant had audiometer measurements that indicated severe hearing impairment (hearing threshold for all subjects >70 dB based on guidelines published by the British Society of Audiology) or were cognitively impaired (25 or higher on MMSE and/or 26 or higher on Ravens Coloured Progressive Matrices).

#### 2.2 Ethics statement

The study was approved by the Cambridge Psychology Research Ethics Committee. All participants gave written informed consent prior to participation, and were compensated for their participation according to the time they spent on the study.

### 2.3 Stimuli

fMRI scanning occurred while subjects were listening to spoken sentences described previously (see Tyler et al. (2011) for further details). The test stimuli in both the *task* and *natural listening* fMRI experiments consisted of normal sentences (*n*= 84), half of which included a local syntactically ambiguous central phrase (e.g. “…*landing planes…*”), a naturally occurring phenomenon in human language and one which does not induce syntactic violations. Even though both interpretations of the ambiguous central phrase are grammatically acceptable, one is encountered more frequently in language (dominant: “*landing planes is*…”) than the other (subordinate: *“landing planes are…”*). When the ambiguous central phrase is followed by a continuation that is inconsistent with its dominant interpretation (e.g. “*landing planes are very noisy”*, indicating that it is the *planes* that are noisy), this interpretation is revised in favour of the alternative subordinate interpretation. Thus, the difference in response to dominant versus subordinate sentences formed the critical comparison for syntactic comprehension. We included 42 sentences matched in syntactic structure to the ambiguous sentences, in which the phrase was unambiguous, and two types of control stimuli: (a) filler sentences (*n*=126) which did not involve the syntactically ambiguous central phrase, but instead consisted of natural sentences matched for length and were included to avoid participants becoming aware of the syntactic manipulations, and (b) non-linguistic baseline items (*n*=42) consisting of acoustic stimuli which were constructed to share the complex auditory properties of speech without triggering phonetic interpretation, and consisted of envelope-shaped ‘musical rain’ (Uppenkamp, Johnsrude, Norris, Marslen-Wilson, & Patterson, 2006) in which the long-term spectrotemporal distribution of energy is matched to that of the corresponding speech stimuli. The contrast between these stimuli and the spoken sentences enabled us to assess whether participants showed intact speech processing abilities in case they did not show sensitivity to syntax.

In both the *task* and *natural listening* studies we assessed each subject׳s sensitivity to syntax by means of an acceptability judgement task in which we asked participants to judge whether each sentence was an acceptable sentence of English. Previous studies have shown that performance in this task provides a measure of participants׳ sensitivity to syntactic structure during sentence processing (Papoutsi et al., 2011, Tyler et al., 2013, Tyler et al., 2011). In the n*atural listening* study the behavioural data was obtained after scanning whereas in the *task* study the data were obtained during the scanning session. In the *natural listening* condition, in order to avoid task-related activation and maximise natural listening, participants listened to the sentences in the scanner without carrying out any task. Approximately 1 month after scanning they carried out the acceptability judgement task on the same sentences. In the *task* condition, participants carried out the acceptability judgement task *during* scanning.

### 2.4 MRI acquisition and preprocessing

Functional, T2^*^-weighted echo-planar images (EPI) were acquired using a Siemens 3T Tim Trio MRI scanner (Siemens Medical Solutions, Camberley, UK) at the MRC Cognition and Brain Sciences Unit, Cambridge, UK. Functional images comprised of 32 oblique axial slices, 3 mm thick (0.75 mm inter-slice gap), angled away from the eyes with in-plane resolution of 3×3 mm^2^ (FOV=192×192 mm^2^, TR/TE=2000/30 ms, flip angle=78°). We also acquired T1-weighted structural images at 1 mm isotropic resolution in the sagittal plane, using an MPRAGE sequence (TR/TI/TE=2250/900/2.99 ms, flip angle=9°). The preprocessing of the functional MRI data was the same as reported in Tyler et al. (2011) comprising of realignment, spatial normalisation and spatial smoothing. Images were slice-time and movement corrected, spatially normalised to the ICBM152 T1 template using unified segmentation (Ashburner & Friston, 2005) and spatially smoothed using an isotropic 8 mm FWHM Gaussian filter in SPM8 (Wellcome Institute of Imaging Neuroscience, London, UK). GM segments were further normalised to a population template generated from the complete image set using a diffeomorphic registration algorithm (DARTEL; Ashburner, 2007), and smoothed using an isotropic Gaussian kernel of 12 mm FWHM. This non-linear warping technique minimises structural variation between subjects and has been shown to perform particularly well in aging populations (Peelle, Cusack, & Henson, 2012).

### 2.5 ICA

**Fig. 1:**
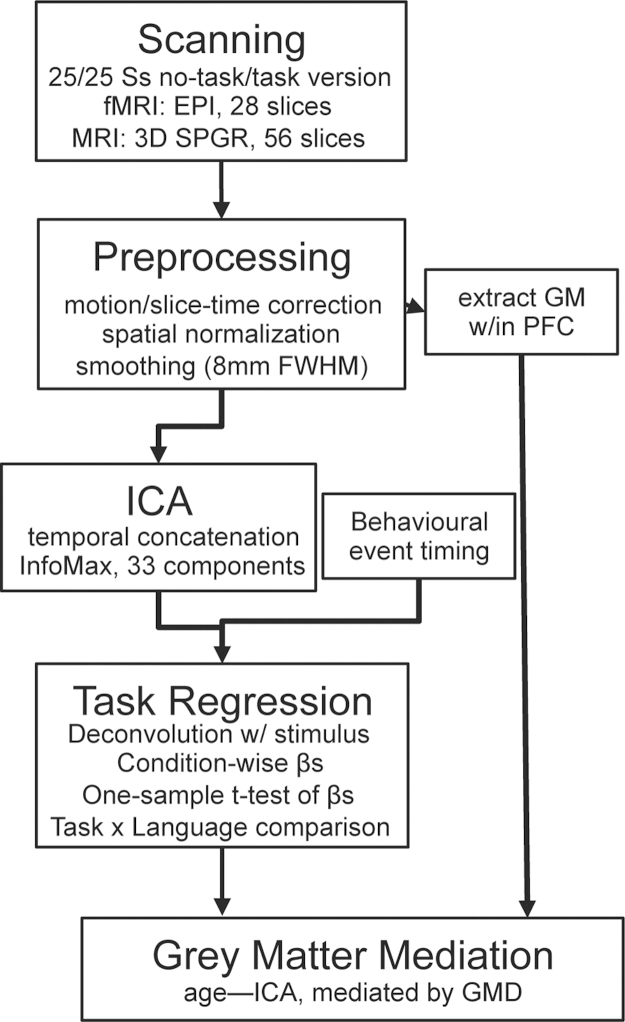
*Schematic of Group ICA analysis pipeline*. Boxes indicate major steps in the analysis pipeline, particular choices for the analysis presented here underneath.

The ICA analysis was used to identify distinct and overlapping networks involved in the *task* and *natural listening* experiments, unbiased with respect to an underlying temporal model for conventional analyses on the *natural listening* data using the general linear model (see Tyler et al., 2011). These spatially independent networks were identified with ICA as implemented in the Group ICA of fMRI Toolbox (GIFT; Calhoun et al., 2001). The general methodology, described in the schematic outlined in Fig. 1, involves performing ICA on functional data concatenated over every participant, creating a series of spatial maps and associated time courses for the group. Group ICA requires that all participants are analysed at once, and a principal component analysis (PCA) was used for compression to allow the datasets from the *task* and *natural listening* experiments to be processed together. In the PCA step, data from all participants for both the *task* and *natural listening* conditions were reduced from the number of time points within the experiment (*n*=355) to the number of dimensions that were estimated in this study to the minimum description length (MDL) criteria for this dataset (Li, Adali, & Calhoun, 2007), which describes a method of reducing the stochastic complexity in a dataset, such that any regularity in the data can be used to compress the data. The stability of the MDL was tested with the software package ICASSO (Himberg, Hyvarinen, & Esposito, 2004), repeating the model estimation 10 times and resulted in a consistent local minimum of 33 principle components.

### 2.6 Component identification

Components with time courses related to the main experimental variables were identified by one-sample *t*-tests of mean ICA activity using the Event Averaging feature of the GIFT toolbox. We first created stick functions representing events of zero duration at the onset of the disambiguating verb following the ambiguous central phrase in the ambiguous sentences (e.g. “*is*” in the sentence “…*hunting eagles is…”)* or the matched location in the unambiguous sentences. We then created stimulus regressors in SPM8 by convolving stimuli functions with the canonical hemodynamic response. Six task conditions were modelled along with the standard set of motion regressors (i.e. *x*/*y*/*z* translation, pitch, roll, and yaw): the syntactically ambiguous conditions (subordinate and dominant sentences), the matched syntactically unambiguous sentences, filler sentences, unacceptable sentences (only in the task condition) and the auditory baseline condition. Each task-related and motion regressor was used to predict the timecourse of each of the 33 components via linear regression; this process is equivalent to standard voxel-wise modelling of the same design matrix, and both massively decreases the number of comparisons being made on the same data, as well as implicitly removing noise components unrelated to the task. One sample *t*-tests on *β* values for each task-related condition for all components were used to select a subset of components for further analysis if they were significant (*p*<0.01) for any of the six conditions of interest, consistent with previous event-related ICA analyses using similar methodology (St Jacques, Kragel, & Rubin, 2011). Loading parameters (or ‘values’) from surviving components were then tested for effects of Task (task versus natural listening), Language (syntactically subordinate versus dominant sentences), and Age, with continuous relationships between network activity and Age being tested via Pearson׳s correlations (*p*<0.05, Bonferroni-corrected for the number of significant components).

### 2.7 Analysis of GM volume

To prefigure the results, only one component showed age-related change. In order to assess whether age effects in network activity may be in response to a fundamental change in brain structure, we performed an additional mediation analysis (Baron & Kenny, 1986) of GM density (GMD) and used this information to reassess the age-related relationships between age (the predictor variable) and ICA activity in the bilateral PFC network (the outcome variable). Mediation is appropriate in this context because it allows us to test for the possibility that any age-related increases in network activity may be at least partially caused by subjects׳ declining grey matter health. Voxel-wise mediation was performed using the M-MEPM toolbox (Wager et al. 2009). Briefly, mediation analysis can be conceptualised as a series of three separate regression equations testing different components of the mediation hypothesis in each voxel within: 1) the age-related decline in GMD (the *a* effect), 2) the relationship between GMD and ICA loading on the bilateral PFC network, controlling for age (the *b* effect) and 3) the extent to which the GMD in a given voxel explains a significant amount of the age-ICA covariance (*ab* effect). We restrict our results to clusters demonstrating a significant effect (*p*<0.01, 15 contiguous voxels) across all paths in the model (conjunction of *a*, *b*, and *ab* effects).

## 3 Results

### 3.1 Behavioural data

Previous studies have shown that a reliable behavioural measure of sensitivity to syntactic structure while comprehending a sentence is a larger number of unacceptable judgements for subordinate compared to dominant sentences (Tyler and Marslen-Wilson, 2008, Tyler et al., 2011) as reflected in a positive difference score. Performance on the acceptability judgement task was collected both in the scanner [*task* condition] and in the behavioural session that followed the scanning session [*natural listening* condition]. These data are reported in Table 1. As predicted, in the *natural listening* condition participants made a large percentage of unacceptable judgements (39.7%) when the continuation was consistent with the less frequent subordinate reading, and a small percentage when it was consistent with the dominant reading (6.4%; paired *t*_24_=10.14, *p*< 0.001). Participants in the *task* condition showed a similar difference between the two conditions (46.0% for subordinate and 11.3% for dominant; (paired *t*_24_=9.53, *p*<0.001)). Together these two results suggest participants were sensitive to syntax as revealed by the syntactic dominance manipulation. We tested for age-related changes in acceptability judgements using Pearson׳s correlation; again, there was no relationship between age and acceptability ratings in either the *task* or *natural listening* condition (*r*_25_=0.11, *r*_25_=0.06, respectively). Furthermore, a moderator analysis on all participants combined (with age as the predictor, acceptability rating as the outcome variable, and experimental condition (task and natural listening) as the moderator) confirmed non-significant effects of Age (*t*_46_=1.29, *p*=0.20) and Age×Task interaction (*t*_46_=−0.84, *p*=0.41) on sentence acceptability ratings. Together, these results confirm that participants of all ages showed sensitivity to the syntactic manipulations, and that there were no age-related changes in these effects. This confirms previous findings of an absence of age-related changes in syntactic comprehension (Tyler et al., 2010), and furthermore suggests that any age-related changes in fMRI activity are related to a fundamental difference in neural activity, and not differences in task performance.

**Table 1:** Participant demographics and behavioural performance.

					Acceptability ratings					
	Age		MMSE		Subordinate		Dominant		Unambiguous	
	*M*	SE	*M*	SE	*M*	SE	*M*	SE	*M*	SE
Natural listening *(post-test)*	53.75	4.33	29.1	0.18	0.39	0.04	0.07	0.01	0.02	0.01
Task *(during scan)*	51.18	3.60	29.0	0.18	0.46	0.04	0.11	0.02	0.05	0.01

### 3.2 ICA: effects of Task, Language and Age

**Fig. 2:**
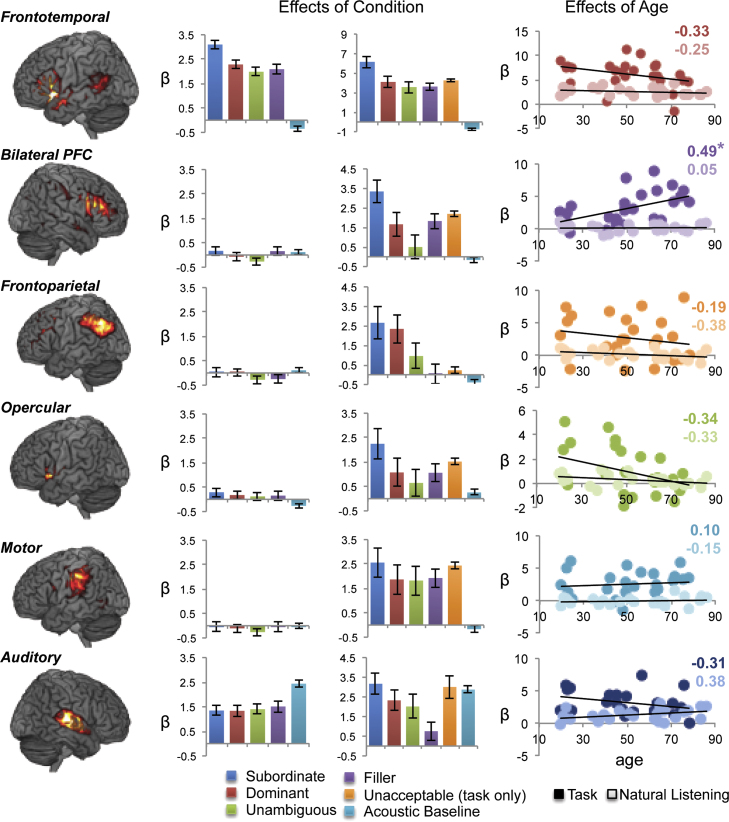
*Effects of Task, Language, and Age on each component*. A) Frontotemporal, bilateral PFC, Frontoparietal, Opercular, Motor, and Auditory components rendered onto a canonical brain. B) Mean activity for syntactically subordinate, dominant, unambiguous, filler sentences, and acoustic baseline. Asterisks indicate significant effects of Language (greater unacceptable judgements for subordinate compared to dominant) for a particular component within each task condition (*task*, *natural listening*; see Table S1C) Age-related correlations across participants (circles; *n*=25 per task/natural listening conditions), using activity values from the syntactically subordinate sentences. Correlation values (Pearson׳s *r*) are displayed separately for each experiment (bold: *task*; light: *natural listening*). Activation is displayed at FWE corrected *p*<0.0001, 25 voxels (*t*>7.9); ^*^−*p*<0.05; ^**^−*p*<0.01.

We used ICA in order to identify reliable networks activated during both naturalistic and experimental language conditions, and to investigate the potential interactions in networks sensitive to syntactic complexity. ICA identified six event-related components that were significantly related (*p*>0.01) to at least one of the event regressors [subordinate, dominant, unambiguous, filler, unacceptable sentences, and acoustic baseline (see Section 2.2)], which were subjected to further examination. These included: 1) a largely left-lateralised Frontotemporal^1^ network comprising the left middle temporal gyrus, inferior frontal gyrus (predominantly left BA 45), anterior cingulate, and much smaller activations in right IFG (BA 45) and MTG (BA22), 2) a right-lateralised Frontoparietal network, comprising a large cluster with peaks in the inferior frontal gyrus (largely BA 45), anterior cingulate cortex and the superior frontal gyrus, bilateral inferior parietal lobule, and the left lobe of the cerebellum, 3) an Opercular network comprising bilateral anterior insula and anterior cingulate cortex, 4) a bilateral PFC network comprising a broad, symmetrical pattern in inferior and middle frontal gyri, 5) a left-lateralised motor cortex component, and 6) a component comprising bilateral auditory cortex. These networks and their component loadings on the five sets of sentence stimuli (+ acoustic baseline) are shown in Fig. 2A and B (see Appendix A, Appendix A for activation peaks and spatial distributions, respectively).

To assess the relevance of the experimental manipulations [Task (task/natural listening) and Language (subordinate/dominant)] to network activity in the six neurocognitive networks we ran a 2×2 multivariate analysis of variance (MANOVA). Only the two types of ambiguous sentences were used in this analysis. The two-way MANOVA indicated significant main effects of the Task manipulation (task, natural listening conditions) in five of the six task-related networks (*F*_1,49_ >4, Table 2) while the main effect of syntactic manipulation (subordinate and dominant sentences) was significant only in Frontotemporal and bilateral PFC networks (*F*_1,49_=9.41, *p*=0.003; *F*_1,59_=6.17, *p*=0.015, respectively), but not any other network. We observed a significant interaction between Language and Task manipulations in the bilateral PFC network (*F*_1,49_=4.10, *p*=0.046), suggesting that this region showed a reliable difference between subordinate and dominant sentences only during the *task* condition. Planned *t*-tests between subordinate and dominant sentences in both *task* and *natural listening* conditions revealed significantly greater network activity for subordinate than dominant sentences in three of the four networks in the *task* condition (*t*_1,24_=7.15, 4.89, and 2.37, for Frontotemporal, bilateral PFC, and Opercular networks, respectively, all *p*<0.05), but *only* the Frontotemporal network showed a significant effect (*t*_1,24_=2.98, *p*<0.05) in the *natural listening* condition (see Fig. 2). Thus, comprehending language while performing a linguistic task appears to recruit additional activity in a trio of task-related networks; which are spatially and temporally independent from activity elicited during merely listening to and comprehending the same sentences.

**Table 2:** Main effects and interactions of Task and Language condition in six task-related networks supporting the processing of ambiguous sentences.

	Task		Language condition		Task×Language	
Component	*F*	*p*	*F*	*p*	*F*	*p*
*Frontotemporal*	86.31	<0.001	9.41	0.003	3.33	0.071
*Bilateral PFC*	43.47	<0.001	6.17	0.015	4.10	0.046
*L FPN*	27.95	<0.001	0.22	0.639	0.04	0.841
*Opercular*	3.24	0.075	1.22	0.272	0.45	0.502
*Motor*	64.34	0.001	1.73	0.191	1.22	0.271
*Auditory*	46.17	<0.001	1.56	0.214	1.65	0.203

The results above describe an intuitively simple yet empirically fundamental result: when participants hear the same linguistic stimuli, but under varying demand characteristics—naturalistic listening versus an experimental task paradigm—they express new networks specifically associated with the experimental task paradigm. Our next question was whether these task-related networks are more susceptible to the effects of age. To do this we looked at the effects of age using Pearson׳s correlations across the entire range of ages in the two independently collected datasets. For this correlation analysis we used individual participants׳ network activity in the subordinate sentence condition, which showed reliably positive activity in all four networks (Fig. 2B; *t*_50_>2.3, *p*<0.01 for all networks). Subsequent correlations between age and network activity demonstrated that only the bilateral PFC network showed a significant *positive* correlation with age (*r*_25_=0.49, *p*=0.01) and this was *only in the task condition* (Fig. 2C, leftmost and rightmost scatterplots), in which subjects made an overt response to each trial. Furthermore, we tested the possibility that this age-related change may be due to low-level perceptual deficits that are synonymous with age. The critical comparison (e.g. the comparison between equal numbers of dominant versus subordinate sentences) relied on a syntactic manipulation that only required subjects to distinguish “is” and “are” (or “was” and “were”), which we chose because they are all relatively low-pitched voiced phonemes and should be robust to high-frequency hearing loss. Nonetheless, the role of age-related sensory deficits in higher cognition is still not completely understood (Schneider, Daneman, & Pichora-Fuller, 2002). Removing the variance associated with hearing ability (average audiometer ratings) did not significantly attenuate this correlation: (Spearman׳s *ρ*_25_=0.46, *p*=0.022; Fisher *z*-test of difference between correlations: *z*=0.13, *p*=0.45), even though audiometer scores showed a significant age-related decline (left: *r*=0.58; right: *r*=0.64; average: *r*=0.63, all *p*<0.01). This result suggests a pattern of bilateral activity in the middle frontal gyrus—well established in both empirical (Davis, Kragel, Madden, & Cabeza, 2011) and meta-analytic investigations of ageing (Spreng et al., 2010)—which shows a reliable age-related increase only during task-related sentential processing, and not during more naturalistic sentence processing, and unrelated to age-related sensory deficits.

### 3.3 Role of GMD in mediating age-related overactivation

**Fig. 3:**
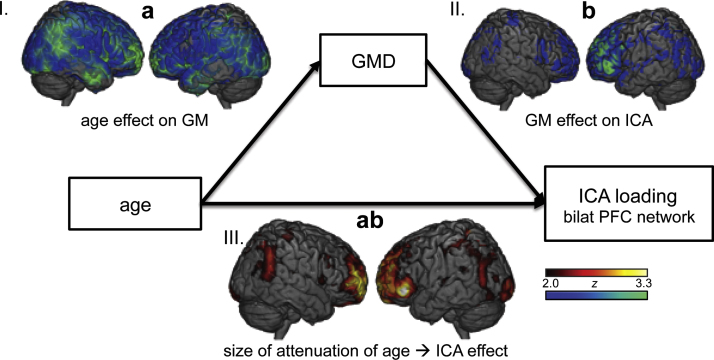
*Results of the mediation of the age-related increase in bilateral PFC network expression by cortical grey matter*. In this model we seek to explain the significant age-related increase in an ICA network comprising bilateral PFC (See Fig. 2C) by proposing grey matter density as a potential mediator. Significant voxels demonstrate either I) the significant relationship between age and grey matter density (*a effect*), II) the independent relationship between GMD and ICA network expression (*b effect*), or III) the attenuation of the age-network expression relationship by GMD (*ab effect*). Significant clusters demonstrating an *ab effect* (which overlap considerably with the same regions demonstrating age-related increases in activity) therefore help to account for older adults׳ increases task-related activity. Colours are represented from *z*=1.96 (*p*=0.05) to *z*=3.29 (*p*=0.001); for cluster peaks and *z*-scores, see Table 3. GMD: grey matter density; ICA: independent component analysis. (For interpretation of the references to colour in this figure, the reader is referred to the web version of this article.)

The above results raise a question of what factors drive the age-related increase in bilateral PFC recruitment: if age-related changes in task-related network activity reflect a response to PFC declines, activity in the driving network should be most influenced by physiological changes (e.g. grey matter density). Such a hypothesis is consistent with the observation that the regions that show the most consistent regional atrophy are the same regions that show increased regional activation in older adults (Greenwood, 2007), and that this paradox is evidence of the functional plasticity of these PFC regions. Supporting this view, we found that a number of PFC clusters partially mediated the age-related increase in bilateral PFC activity we previously identified during the task condition. Fig. 3 depicts the A–B–C mediation model, and maps of significant *a*, *b*, and *ab* effects (I, II, and III, respectively). As expected, linear effects of age on GMD (*a effect*) were widespread across all regions of cortex (see Table 3 for cluster information). More interestingly, a widespread pattern of PFC and dorsal parietal regions showed a significant negative relationship between GMD and ICA loading, when controlling for the effects of age (*b effect*). The fact that many of these regions overlap with the activity map of the bilateral PFC component (see Fig. 2, S1) suggests that reduced local GMD promotes greater bilateral PFC network activity (Meunier, Stamatakis, & Tyler, 2014). Third, a largely overlapping set of PFC and parietal regions demonstrated an independent role in attenuating the age-ICA relationship (*ab effect*). Regions that showed evidence for all three effects are the primary evidence for GM-mediated explanation for bilateral PFC increases; these include bilateral MFG, bilateral superior frontal gyrus, and bilateral operculum (see Table 3, Fig. 3). Thus, many of the regions that demonstrate an age-related increase in network expression are the same regions in which reductions in grey matter accounts for this functional increase. This result suggests another potential explanation for age-related increases in PFC activity unrelated to syntactic processing.

**Table 3:** MNI coordinates for significant mediation clusters.

Region	BA	*x*	*y*	*z*	*Z*_ab_	Voxels
L superior frontal gyrus	11	−10	58	−26	5.15	42
R superior frontal gyrus	10	24	60	−4	5.64	283
L inferior frontal gyrus	47	−52	34	−4	5.23	100
R lingual gyrus	17	22	−90	−4	4.93	16
L superior frontal gyrus	10	−36	48	18	4.85	34
L superior frontal gyrus	10	−14	64	22	5.23	50
R inferior parietal lobule	40	48	−50	34	5.15	44
L precuneus	7	−28	−54	52	5.05	33

*Note*: All clusters thresholded at voxel-level *p*<0.01, extent threshold 15 voxels. BA – Brodmann area; *Z*_ab_ – attenuation of the direct effect (age→ICA) resulting from the inclusion of the mediator.

## 4 Discussion

The goal of the present study was to determine whether there are age-related changes in the relationship between task-related neural networks and networks intrinsic to natural language comprehension. We found minimal age-related changes in the FT language network that is essential for syntactic analysis during language comprehension, but significant age-related differences in activity in other networks when we introduced a task that was not essential to natural language comprehension. Age-related changes in network expression were only observed under the task condition, and were confined to the bilateral PFC network. Furthermore, we found that the age-related increase in bilateral frontal network activity was mediated by GM density in a number of prefrontal regions, suggesting that age related differences in task-related activity might also emerge due to grey matter declines. Together these results help to explain both the stability of the frontotemporal language system across the adult lifespan, as well as revealing how the addition of task-related operations may engender patterns of age-related changes in brain activity, which in turn may confuse our interpretation of ageing effects.

### 4.1 Performing an explicit task versus listening to sentences

By using an experimental procedure that explicitly manipulated both linguistic and non-linguistic processing demands, we were able to model activation differences attributable to task-related activation that is independent of the linguistic manipulations of interest (Wright et al., 2011). We found that one network was consistently observed in both the *task* and *natural listening* conditions—a distributed, primarily left-lateralised, network comprising left inferior frontal and bilateral middle temporal cortex (Fig. 2B). This Frontotemporal network was associated with the predicted pattern of syntactic performance in which participants rated syntactically ambiguous sentences followed by a subordinate continuation to be unacceptable, and those followed by a dominant continuation to be acceptable. This is because for subordinate continuations, the preferred syntactic representation (the dominant reading) must be overturned since it is inconsistent with the disambiguating verb (“…bullying teenagers IS…”), whereas for dominant sentences, the disambiguating verb is consistent with the preferred syntactic interpretation (“…bullying teenagers ARE…”) that listeners construct on-line as they hear the ambiguous central phrase (Mason et al., 2003, Rodd et al., 2010). This is also consistent with previous findings showing that syntactic processing is typically associated with a functionally connected left BA45 and left middle temporal gyrus network. Damage to either left frontal or temporal regions (or the connections between them) typically results in syntactic deficits (Papoutsi et al., 2011, Tyler et al., 2011), whereas damage to comparable regions in the RH does not. In follow-up analyses carried out on the task and natural listening datasets separately we found that the weak RH activity we observed in frontotemporal regions within the language network was only present in the task data and not the natural listening data, confirming previous studies using the same stimuli (Tyler et al, 2011). Our finding here, that the LH Frontotemporal network is engaged during the computation of syntactic analysis when there is no concomitant task supports its role in forming the core syntactic processing network in spoken language (Tyler & Marslen-Wilson, 2008).

In contrast, a set of networks involving more dorsal regions were additionally recruited in the context of a demanding task, suggesting that the demands associated with performing an overt language task generated a number of additional cognitive processes [e.g. strategic retrieval, explicit selection (Thompson-Schill et al., 2005, Ye and Zhou, 2009)] not intrinsic to language processing. For many cognitive operations, the use of an experimental task may indeed be ecologically valid, and age-related differences emerging from these tasks may reflect real-world deficits in cognition. However, syntactic processing may be less amenable to the demand characteristics inherent to some task paradigms. Listeners typically do not explicitly judge the acceptability of sentences during their on-line processing of spoken language in naturalistic situations (Newmeyer, 1983). Thus, our results show that performing a non-intrinsic task while processing spoken sentences elicits activity in a host of additional task-related networks in dorsal frontal and parietal regions largely non-overlapping with the core language network. These task-related networks resemble the Multiple Demand Network (Duncan, 2010), a system of task-related regions which are activated when participants are engaged in complex, sequential, and goal-directed operations.

### 4.2 Age-related changes in network expression

We also found that there were no age-related changes in the expression of the language network during the more naturalistic language processing (*natural listening)* context, consistent with previous reports on the stability of this network across the lifespan (Antonenko et al., 2013, Peelle et al., 2010). The Task×Language condition interaction in the bilateral PFC network, and subsequent age-related correlation (Fig. 2C), indicates that age-related differences in network expression emerged only when participants were performing a task. Furthermore, these age-related differences in neural activation were observed in the absence of any behavioural differences between younger and older adults in either the *task* or *natural listening* conditions. This set of circumstances would normally be thought of as evidence of compensation – i.e. more activity and preserved cognition. However, because these age-related differences in network expression were absent during the natural listening condition, it may be reasonable to conclude that these “compensatory” activations are simply a consequence of the experimental task demands engendered by the task condition in our study (Schneider-Garces et al., 2010). Although this finding needs to be investigated in other domains, the current data suggests that typical experimental tasks used to assess cognitive ageing may possibly overestimate age-related differences in brain activity by assuming that any additional activated regions in older compared to younger adults implies that the network involved in a cognitive function has changed. Though this potential confound is explicitly addressed in canonical univariate designs with subtractive logic, this brand of logic is nonetheless not infallible and small but significant age-related differences may mask the more robust activation patterns elicited by ICA. Furthermore, it is possible that subtractive logic may sometimes allow researchers to misattribute the effects of age-related changes to changes in some aspects of cognition, whereas they may be due to systemic variations in the reliance on task-related functions, particularly due to cognitive operations that are reliably found to decline with age, including the maintenance of task goals (Clapp et al., 2011, Gold et al., 2010, Sandberg et al., 2013) or working memory capacity (Cappell et al., 2010, McVay and Kane, 2012).

An alternative, but not exclusive explanation for these age-related changes in frontal activity is that they represent the consequence of more fundamental changes in functional and structural network architecture (Antonenko and Floel, 2014, Davis et al., 2012, Persson et al., 2006). One piece of evidence in support of this idea is that we found that prefrontal and parietal grey matter density partially mediated the age-related increase in bilateral PFC expression (Fig. 3). This suggests that age-related increases in PFC activity may be in response to general GM declines (Kalpouzos, Persson, & Nyberg, 2012). The mediation analysis clarifies this in two ways: first, a number of PFC regions showed a negative relationship between GMD and ICA loading (blue regions in Fig. 3 demonstrating the *b effect*), demonstrating that activity was the greatest in regions with the lowest GMD; in other words, the task-related bilateral PFC activity is most prominent in those who need it most (Nyberg et al. 2014). Second, a very similar pattern of PFC voxels partially attenuated the age-ICA relationship (*ab effect*), suggesting that GM loss serves as a trigger for bilateral frontal overactivation (Meunier et al., 2014). Nonetheless, we found no evidence that this pattern of network activity contributed to participants׳ performance on the acceptability judgment task suggesting that the age-related shift towards greater bilateral prefrontal activity may be associated with compensation *attempt*, but not *success* (Cabeza & Dennis, 2011). This may explain the often-observed pattern of bilateral PFC activity (Spreng et al., 2010), which is not always associated with improved cognition. In this respect it is unsurprising that performance on an acceptability task is not improved with additional activity in regions outside the frontotemporal system, which forms the core of language processing *throughout* the lifespan.

In conclusion, the present results reveal the impact of performing a task that is not an intrinsic aspect of language functioning, and provide evidence that these changes may result from the inclusion of attentionally demanding cognitive tasks and their associate response demands. These findings suggest that future ageing studies should account for the unique demand characteristics associated with performing a psychological task.

## Appendix A Supporting information

Supplementary data Fig. S1 *Task-related components supporting language comprehension*. Images are thresholded at FWE corrected *p*<0.0001, 25 voxels.

## Footnotes

1 Hereafter we use the capitalised “Frontotemporal” to denote the specific network in this study.

Appendix A Supplementary data associated with this article can be found in the online version at doi:10.1016/j.neuropsychologia.2014.08.017.
