# Supplementary figures and images for: Age-related sensitivity to task-related modulation of language-processing networks
Source: Neuropsychologia. 2014 Oct;63:107–15. doi: 10.1016/j.neuropsychologia.2014.08.017 (PMC4410794; doi:10.1016/j.neuropsychologia.2014.08.017)

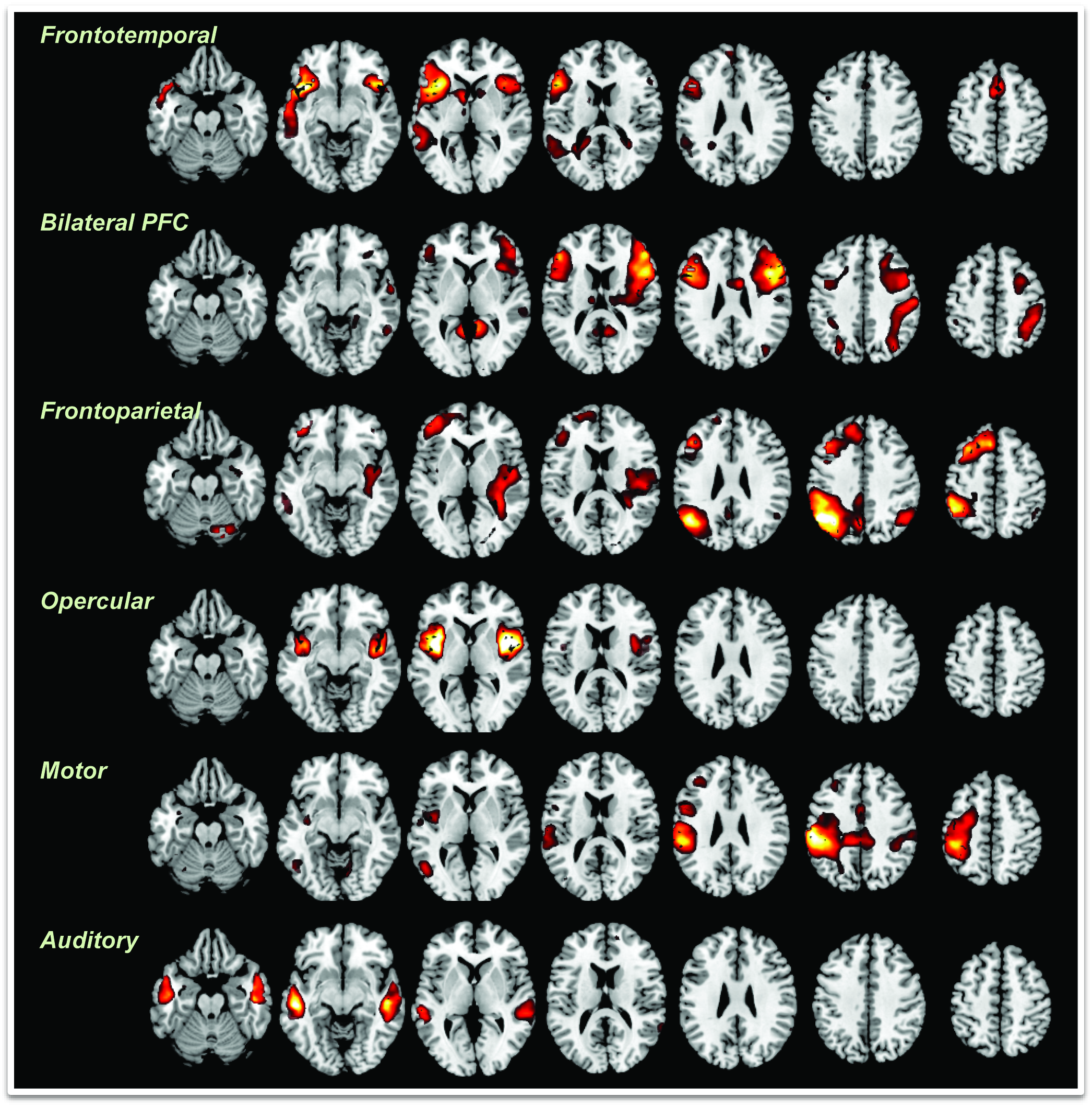

Supplement: Supplementary file 1 — Supplementary data Fig. S1 Task-related components supporting language comprehension. Images are thresholded at FWE corrected p<0.0001, 25 voxels. [file mmc1.zip › FigureS1.tif]
