# Supplementary material for: Age-related sensitivity to task-related modulation of language-processing networks
Source: Neuropsychologia. 2014 Oct;63:107–15. doi: 10.1016/j.neuropsychologia.2014.08.017 (PMC4410794; doi:10.1016/j.neuropsychologia.2014.08.017)
Supplement: Supplementary file 2 — Supplementary data [file mmc2.doc]

Table S1. **MNI coordinates for significant ICA components.**

| **Region** | **BA** | **x** | **y** | **z** | **t** | **voxels** |
| --- | --- | --- | --- | --- | --- | --- |
| ***Auditory Network*** |  |  |  |  |  |  |
| L Superior temporal gyrus | 41/42 | -48 | -18 | 6 | 44.18 | 1892 |
| R Superior temporal gyrus | 41/42 | 48 | -21 | 9 | 40.42 | 1915 |
|  |  |  |  |  |  |  |
| ***Frontotemporal Network*** |  |  |  |  |  |  |
| L Inferior frontal gyrus | 45 | -54 | 27 | 6 | 29.70 | 5601 |
| *L Middle temporal gyrus* | 21 | -57 | -12 | -12 | 27.80 |  |
| *L IFG* | 45 | -48 | 30 | 0 | 26.92 |  |
| Middle cingulate cortex | 6/24 | -3 | 9 | 63 | 24.49 | 306 |
| R Middle temporal gyrus | 21 | 54 | -33 | 0 | 24.04 | 990 |
| Anterior cingulate cortex | 32 | -9 | 54 | 27 | 18.85 | 185 |
| R Inferior frontal gyrus | 47/45 | 51 | 24 | -3 | 17.27 | 65 |
|  |  |  |  |  |  |  |
| ***Motor Network*** |  |  |  |  |  |  |
| L Motor cortex | 4 | -51 | -24 | 45 | 25.90 | 5251 |
|  |  |  |  |  |  |  |
| ***Opercular Network*** |  |  |  |  |  |  |
| R Insula | 13/14 | 39 | 18 | -12 | 43.02 | 1988 |
| L Insula | 13/14 | -42 | 15 | -9 | 40.90 |  |
| Anterior cingulate cortex | 24 | 0 | 18 | 39 | 35.24 | 1686 |
|  |  |  |  |  |  |  |
| ***Frontoparietal Network*** |  |  |  |  |  |  |
| Anterior cingulate cortex | 32 | 9 | 30 | 51 | 30.43 | 2585 |
| *Superior frontal gyrus* | 9 | 15 | 35 | 48 | 27.17 |  |
| R Inferior parietal lobule | 40 | 54 | -51 | 42 | 29.37 | 844 |
| L Cerebellum |  | -33 | -72 | -42 | 26.89 | 658 |
| L Inferior parietal lobule | 40 | -51 | -51 | 48 | 20.00 | 158 |
|  |  |  |  |  |  |  |
| ***Bilateral Frontal Network*** |  |  |  |  |  |  |
| R Middle frontal gyrus | 46 | 45 | 15 | 30 | 31.65 | 1495 |
| L Middle frontal gyrus | 46 | -45 | 18 | 27 | 28.57 | 1297 |

Note: All clusters thresholded at voxel-level p < 0.0001 FDR-corrected, extent threshold 25 voxels. Regions in italics indicate sub-peaks of larger clusters. BA – Brodmann area.
